# Rare variants and loci for age-related macular degeneration in the Ohio and Indiana Amish

**DOI:** 10.1007/s00439-019-02050-4

**Published:** 2019-07-31

**Authors:** Andrea R. Waksmunski, Robert P. Igo, Yeunjoo E. Song, Jessica N. Cooke Bailey, Renee Laux, Denise Fuzzell, Sarada Fuzzell, Larry D. Adams, Laura Caywood, Michael Prough, Dwight Stambolian, William K. Scott, Margaret A. Pericak-Vance, Jonathan L. Haines

**Affiliations:** 1 Department of Genetics and Genome Sciences, Case Western Reserve University, Cleveland, OH USA; 2 Cleveland Institute for Computational Biology, Case Western Reserve University, Cleveland, OH USA; 3 Department of Population and Quantitative Health Sciences, Case Western Reserve University, Cleveland, OH USA; 4 John P. Hussman Institute for Human Genomics, University of Miami Miller School of Medicine, Miami, FL USA; 5 Department of Ophthalmology, University of Pennsylvania, Philadelphia, PA USA

## Abstract

Age-related macular degeneration (AMD) is a leading cause of blindness in the world. While dozens of independent genomic variants are associated with AMD, about one-third of AMD heritability is still unexplained. To identify novel variants and loci for AMD, we analyzed Illumina HumanExome chip data from 87 Amish individuals with early or late AMD, 79 unaffected Amish individuals, and 15 related Amish individuals with unknown AMD affection status. We retained 37,428 polymorphic autosomal variants across 175 samples for association and linkage analyses. After correcting for multiple testing (*n* = 37,428), we identified four variants significantly associated with AMD: rs200437673 (*LCN9*, *p *= 1.50 × 10^−11^), rs151214675 (*RTEL1*, *p *= 3.18 × 10^−8^), rs140250387 (*DLGAP1*, *p *= 4.49 × 10^−7^), and rs115333865 (*CGRRF1*, *p *= 1.05 × 10^−6^). These variants have not been previously associated with AMD and are not in linkage disequilibrium with the 52 known AMD-associated variants reported by the International AMD Genomics Consortium based on physical distance. Genome-wide significant linkage peaks were observed on chromosomes 8q21.11–q21.13 (maximum recessive HLOD = 4.03) and 18q21.2–21.32 (maximum dominant HLOD = 3.87; maximum recessive HLOD = 4.27). These loci do not overlap with loci previously linked to AMD. Through gene ontology enrichment analysis with ClueGO in Cytoscape, we determined that several genes in the 1-HLOD support interval of the chromosome 8 locus are involved in fatty acid binding and triglyceride catabolic processes, and the 1-HLOD support interval of the linkage region on chromosome 18 is enriched in genes that participate in serine-type endopeptidase inhibitor activity and the positive regulation of epithelial to mesenchymal transition. These results nominate novel variants and loci for AMD that require further investigation.

**Electronic supplementary material:**

The online version of this article (10.1007/s00439-019-02050-4) contains supplementary material, which is available to authorized users.

## Introduction

Age-related macular degeneration (AMD) is the third leading cause of vision loss in the world (Ayoub and Patel 2009). It is characterized by the deterioration of the central field of vision due to the accumulation of lipid deposits (drusen), inflammation, and neurodegeneration in the macula (Fritsche et al. 2014). AMD is a multifactorial disease with numerous influential genetic and environmental risk factors. Environmental factors constitute about 20–40% of total AMD variance and include age, smoking, hypertension, and diet (Fritsche et al. 2014; Seddon 2013). More than half of AMD heritability is explained by 52 independent common and rare genetic variants across 34 genomic loci (Fritsche et al. 2016). Therefore, about half of AMD heritability is unaccounted for by known genetic polymorphisms and may be partially resolved with the identification of rare variants (Manolio et al. 2009).

As the minor allele frequency (MAF) for a variant decreases, the sample size required to detect an association increases linearly with 1/MAF (Manolio et al. 2009). Consequently, traditional genome-wide-association studies (GWAS) have been largely unable to detect associations with rarer variation (typically MAF < 0.01) unless the sample size in is the tens of thousands. In contrast, GWAS on a sample of large families can increase the power to detect such variants through enrichment of rare variants segregating in families with many affected members (Auer and Lettre 2015). Isolated founder populations, like the Amish, can amplify the power of family-based studies through sharing of founder variants. The Amish are descendants of Swiss Anabaptists who settled in North America 200–300 years ago and maintained their culturally segregated community for generations by marrying within their faith group and adhering to a uniform, conservative lifestyle (Strauss and Puffenberger 2009). Therefore, they have lower genetic and environmental heterogeneity compared to the general population of European descent. In addition, Amish communities comprise large families with an average of seven to nine children in each nuclear family (McKusick et al. 1964). Extensive genealogical data are available for this community in the Anabaptist Genealogy Database (AGDB) making this a valuable population to identify rare genetic variation by combining the advantages of isolated populations and large pedigrees (Agarwala et al. 2003).

To identify novel variants underlying the pathophysiology of AMD, we performed association and linkage analyses on Illumina HumanExome chip data for 175 related Amish individuals from Ohio and Indiana. We also examined in silico functional annotations of the genes from our linkage regions to determine whether these genes participate in biological processes that may be relevant for AMD pathology.

## Materials and methods

### Study participants

**Fig. 1:**
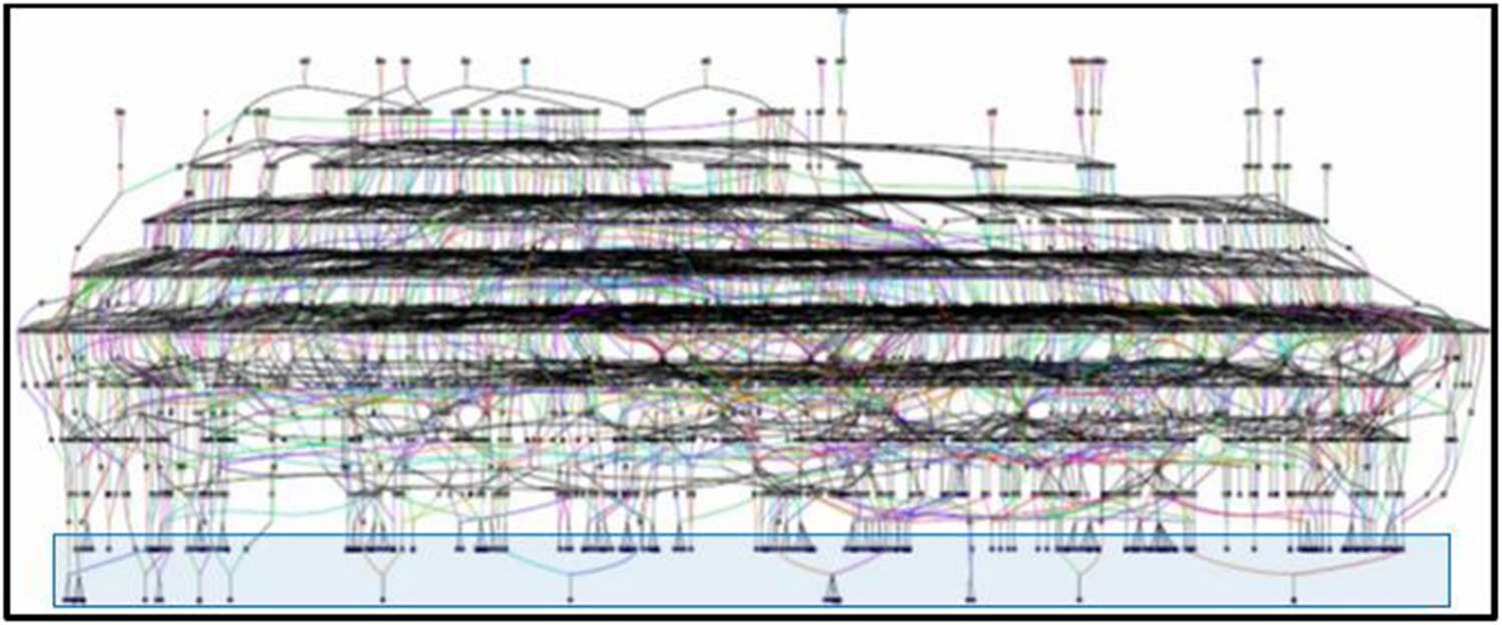
All-connecting path pedigree of the 175 Amish individuals in this study. The pedigree was drawn using information from the Anabaptist Genealogy Database (AGDB). Circles: females. Squares: males. The genotyped individuals are within the shaded blue box

The Amish individuals were identified from published Amish community directories, referral from other Amish community members, or due to close relationships to other research subjects. All study participants were at least 65 years old. The AMD affection status was defined by a self-reported diagnosis on a health questionnaire, which asked whether the individual had ever received an AMD diagnosis from a physician. We previously demonstrated that self-reported AMD statuses have positive and negative predictive values of around 90% in this population (Hoffman et al. 2014). Some Ohio Amish participants also received a clinical exam at the Wooster Eye Clinic in Wooster, Ohio where ocular coherence tomography images were obtained for both eyes. Participants’ eyes were graded on a scale from 0 to 5 using the modified Clinical Age-Related Maculopathy Staging (CARMS) system (Seddon et al. 2006b; Sardell et al. 2016). Individuals with grades 0, 1, and 2 in both eyes were designated as unaffected in this study, and individuals with grades 3–5 in at least one eye were designated as affected. Grades of 3 were indicative of early/intermediate AMD, and grades of 4 or 5 were classified as advanced AMD. For this study, 181 Amish samples included 87 individuals with confirmed or self-reported AMD, 79 unaffected individuals, and 15 related Amish individuals with unknown AMD phenotype. Using the Anabaptist Genealogy Database (Agarwala et al. 2003), we constructed a 2118 person all-connecting path pedigree for the 175 Amish individuals that passed quality control and were closely related (Fig. 1). This included 86 affected with AMD, 77 unaffected individuals, and 12 relatives with an unknown AMD affection status. Informed consent was obtained from all study participants, and the study was approved by the institutional review boards at Vanderbilt University and Case Western Reserve University. Study procedures were performed in accordance with the tenets of the Declaration of Helsinki.

### Genotyping and quality control

A total of 192 samples, which included 181 unique Amish samples, 5 Amish duplicates, and 6 Coriell HapMap trio controls of European descent, were genotyped using the Illumina HumanExome BeadChip v1.1 at the Vanderbilt Technologies for Advanced Genomics (VANTAGE) DNA Resources Core. Genotype data were called and clustered with Illumina’s GenomeStudio (Illumina). The accuracy of genotype calling using GenomeStudio is improved by increasing the sample size (Grove et al. 2013). Therefore, an additional 2280 non-Amish samples that were genotyped by VANTAGE with the same protocols and at the same time as the Amish samples were imported into GenomeStudio and clustered with the Amish samples. The data from these non-Amish samples were not used in the subsequent association and linkage analyses. All samples demonstrated call rates greater than 95% and were used for extensive quality control in GenomeStudio as summarized in Supplemental Fig. 1 and Supplemental Table 1 (Guo et al. 2014). Briefly, we excluded variants for low call rate, GenTrain score for cluster quality, cluster separation, and intensity values (Supplemental Table 1). Hemizygous variants were evaluated as well as variants with Mendelian and replication errors (Supplemental Table 1). Rare variant calling was performed manually with GenomeStudio’s GenCall algorithm and with zCall, a separate rare variant calling algorithm (Goldstein et al. 2012). zCall is utilized as a post-processing step for calling variants with no genotypes assigned after initial variant calling with GenomeStudio’s GenCall algorithm. It uses the intensity profile of the homozygous cluster of the common allele to determine the location of the other homozygous and heterozygous genotype clusters (Goldstein et al. 2012). All variants with at least four new heterozygous calls in zCall were manually reviewed in GenomeStudio.

A total of 146,571 variants and 2466 samples, including 181 Amish individuals, passed quality control in GenomeStudio and were evaluated for sex mismatch, race mismatch, and heterozygosity outliers using PLINK v1.07 (Purcell et al. 2007). Sex mismatched samples were evaluated using heterozygosity rates for the X chromosome and call rates for the Y chromosome (Supplemental Fig. 2). One unresolvable sex mismatch in our Amish samples was identified and removed from the study. An XXY Amish male and an XO Amish female were identified and retained. Ancestry mismatch was assessed using the EIGENSTRAT software package and about 3000 ancestry-informative markers (AIMs) from the exome chip (Price et al. 2006). Principal component analysis (PCA) demonstrated that the combined sample set is multi-ethnic and that the Amish samples clustered together with other Europeans as expected (Supplemental Fig. 3). None of our Amish samples were in the extremes of the heterozygosity distribution calculated by PLINK v1.07 (Purcell et al. 2007), so none of them were excluded as outliers (Supplemental Fig. 4). The heterozygosity rate can indicate problematic, potentially contaminated DNA samples (Guo et al. 2014). Of the 242,901 variants genotyped, 37,428 were polymorphic in the Amish sample, passed extensive quality control, and served as input for our association and linkage analyses. Variants were considered polymorphic if they had a non-zero minor allele frequency calculated by the RObust Association-Detection Test for Related Individuals with Population Substructure (ROADTRIPS) software v2.0. We omitted five Amish individuals from our association and linkage analyses due to excessively distant relatedness in the pedigree (Supplemental Fig. 5). Of the remaining 175 Amish individuals, 86 are affected, 77 are unaffected, and 12 have an unknown AMD status.

### Association analysis

The samples in this study are highly interrelated (Fig. 1). To account for these relationships and population structure, we performed association testing using the ROADTRIPS software v2.0 (Thornton and McPeek 2010). This program determines associations between genotypes and binary traits while adjusting for the relatedness and population structure of the samples. It calculates and uses an empirical covariance matrix based on the genotypes from the genotype data to account for population structure. ROADTRIPS determines three different test statistics for association: the RM, R_χ_, and RW tests (Thornton and McPeek 2010). For each of these tests, *p* values are calculated based on a Chi squared distribution with 1 *df* asymptotic null distribution (Thornton and McPeek 2010). We used the RM test statistic because it allows inclusion of both unaffected individuals and individuals with unknown phenotype in the analysis and is more powerful than the other two when samples are related and pedigree structure is known (Thornton and McPeek 2010). We used kinship coefficients for the 175 Amish individuals calculated by KinInbcoef to account for the pedigree structure in our data (Bourgain 2003). The software program determines the weight of individuals with unknown phenotype in the analysis by a user-defined population prevalence for the trait and the phenotypes of their genotyped relatives (Thornton and McPeek 2010). We set the population prevalence of AMD to 0.13 based on published estimates of its prevalence in individuals of European descent of an age typical of this pedigree (Shalev et al. 2011; Smith et al. 2001).

### Linkage analysis

We performed multipoint linkage analyses on a panel of 5668 autosomal variants chosen to be informative for stretches of identity by descent on the exome chip (Abecasis Lab 2013). The 175 Amish individuals in this study are connected in a multigenerational pedigree of 2118 Amish individuals (Fig. 1). Since analyzing very large, complex pedigrees is computationally intractable, we used PedCut to partition the all-connecting path pedigree into densely affected sub-pedigrees with a maximum bit size of 24 (Liu et al. 2008). Parametric heterogeneity LOD (HLOD) scores were determined with the Multi-Point Engine for Rapid Likelihood Inference (MERLIN) software tool (Abecasis et al. 2002) under affected-only dominant and recessive models. The penetrance values for the dominant model were 0, 0.0001, or 0.0001 for 0, 1, or 2 copies of the disease allele, respectively. Under the recessive model, penetrance values were defined as 0, 0, or 0.0001 for 0, 1, or 2 copies of the disease allele, respectively. We set disease allele frequencies to 0.01 and 0.10 for each of these models. We performed multipoint linkage analyses across all autosomes with HLOD scores evaluated at each marker and at every 1 centimorgan (cM; Haldane) with a bit size threshold of 24. For chromosomes with genome-wide significant multipoint HLOD scores, we repartitioned the all-connecting path pedigree into sub-pedigrees with maximum bit sizes of 23 and 25 to test the robustness of our findings to changes in sub-pedigree construction. The model parameters for these analyses were consistent with those used in the initial multipoint analyses.

For chromosome 1, we performed a multipoint linkage analysis with liability classes for carriers of the *CFH* Y402H (rs1061170) (Edwards et al. 2005; Hageman et al. 2005; Haines et al. 2005; Klein et al. 2005) and P503A (rs570523689) variants (Hoffman et al. 2014). *CFH* Y402H failed quality control in our dataset. Therefore, we identified a surrogate variant (rs1329424, *r*^2^ = 1.0) for *CFH* Y402H using the LDmatrix module of LDlink to compare linkage disequilibrium statistics among *CFH* variants that had passed quality control in our study and the Y402H variant in the CEU population from Phase 3 (Version 5) of the 1000 Genome Project (Machiela and Chanock 2015). We identified *CFH* P503A carriers by performing customized TaqMan genotyping assays of the variant in our Amish cohort (unpublished data). Briefly, P503A genotyping was carried out using a Custom TaqMan SNP Genotyping Assay (Thermo Fisher) to interrogate the presence of a C (P503) or G (A503) at position 1507 in the *CFH* transcript. Assays were carried out per the manufacturer’s instructions using TaqMan Genotyping Master Mix (Thermo Fisher) and 10 ng of genomic DNA per reaction. Reactions were run on a QuantStudio 7 PCR machine, and genotypes were called by the QuantStudio analysis software. In our conditional linkage analysis, penetrance values were 0.13, 0.312, and 0.624 for 0, 1, and 2 copies of the disease allele, respectively, for carriers of only the surrogate for Y402H. We chose these values based on the population prevalence of AMD in the Caucasian population (13%) (Shalev et al. 2011; Smith et al. 2001) and the expected prevalence based on the published odds ratio (OR) for Y402H, which is 2.4 per copy of the risk allele in individuals of European descent (Haines et al. 2005; Sofat et al. 2012). For carriers of only the P503A variant or both variants, penetrance values were 0.13, 0.6, and 0.6 for 0, 1, and 2 copies of the disease allele, respectively. The penetrance value for the P503A risk allele was derived from the number of affected P503A carriers we identified in a previous study (Hoffman et al. 2014). For non-carriers of both variants, penetrance values were consistent with those used for the multipoint linkage analyses of the other autosomes.

### In silico functional analysis of genes from 1-HLOD support intervals of linkage regions

To determine whether genes present in the 1-HLOD support intervals (Supplemental Tables 2–4) of our linkage regions on chromosomes 8 and 18 are functionally related to one another, we extracted gene boundaries from Ensembl (human genome build 37) and performed gene ontology (GO) enrichment analyses with the ClueGO v2.5.3 plug-in (Bindea et al. 2009) of Cytoscape v3.7.1 (https://cytoscape.org/). Specifically, terms from the following classifications in GO were considered: Immune System Process, Biological Process, Molecular Function, and Cellular Component. We included all evidence codes (experimental, non-experimental, author statements from publication, and curator statements) in the analysis. We calculated *p* values using right-sided hypergeometric tests and the Benjamini–Hochberg correction for multiple testing. We chose medium network specificity to identify representative pathways for our genes of interest. This level of specificity examines GO levels 3–8, requires at least three genes per GO term, and ensures that mapped genes represent at least 4% of the total associated genes. We also used a Kappa score of 0.4 for our GO term network connectivity threshold. GO terms were iteratively compared, merged into functional groups, and visualized in Cytoscape.

## Results

### Association analysis

**Fig. 2:**
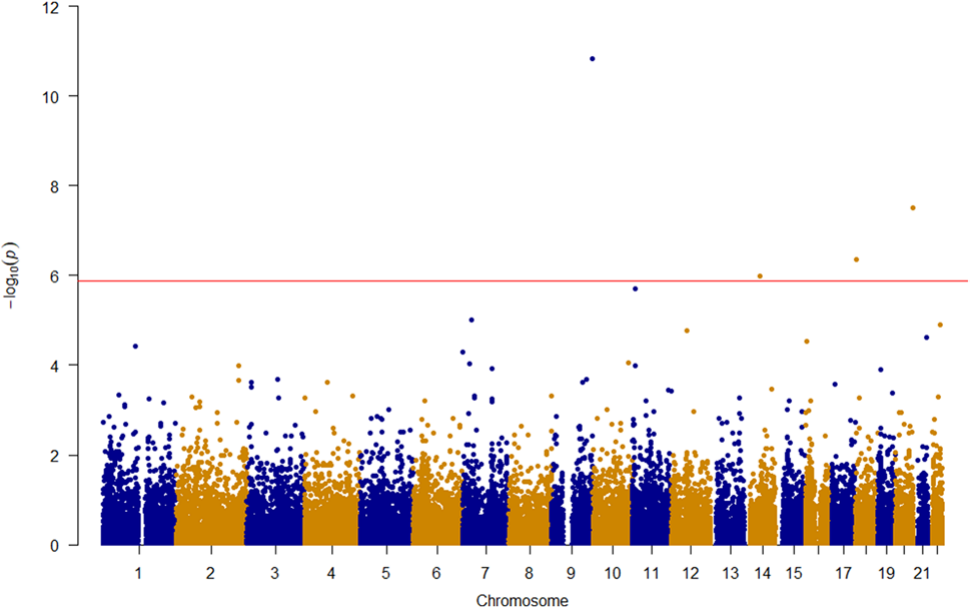
Manhattan plot of *p* values obtained from association testing using ROADTRIPS. The red line denotes the Bonferroni correction threshold for multiple testing (*p* < 1.34 × 10^−6^). Four variants passed this threshold and are summarized in Table 1

Of the 37,428 polymorphic autosomal variants that passed quality control, four variants met the Bonferroni correction threshold (*p* < 1.34 × 10^−6^): rs200437673 (chromosome 9, *LCN9*, *p* = 1.50 × 10^−11^), rs151214675 (chromosome 20, *RTEL1*, *p* = 3.18 × 10^−8^), rs140250387 (chromosome 18, *DLGAP1*, *p* = 4.49 × 10^−7^), and rs115333865 (chromosome 14, *CGRRF1*, *p* = 1.05 × 10^−6^) (Fig. 2; Table 1). *p* values were not corrected for inflation because the genomic control factor was 1.05 (Supplemental Fig. 6). These variants demonstrate novel associations with AMD and do not map to the AMD susceptibility loci identified by the International AMD Genomics Consortium (IAMDGC) based on physical distance (Fritsche et al. 2016). While these variants were identified in the Amish, we found that they are rare to low-frequency (MAF < 0.01) in outbred populations of European descent including the IAMDGC dataset and the gnomAD database (Table 2).

**Table 1:** AMD-associated variants identified with ROADTRIPS testing of Amish families

rsID	Chr.	Position	Alleles	Gene(s)	Consequence	Allele count			*P*
						Affected	Unaffected	Unknowns	
rs200437673	9	138,555,237	A/G	*LCN9*	Missense	5	0	1	1.50 × 10^−11^
rs151214675	20	62,293,235	G/A	*RTEL1* *RTEL1*-*TNFRSF6B*	Missense Non-coding Transcript	6	1	0	3.18 × 10^−8^
rs140250387	18	3,534,424	A/G	*DLGAP1*	Synonymous	3	1	0	4.49 × 10^−7^
rs115333865	14	55,004,449	G/A	*CGRRF1*	Missense	10	2	1	1.05 × 10^−6^

*p* values were obtained from the RM test

Variant positions are given for build 37 (hg19) of the human genome

AMD, age-related macular degeneration; ROADTRIPS, RObust Association-Detection Test for Related Individuals with Population Substructure; Chr., chromosome; *LCN9*, lipocalin 9; *RTEL1*, regulator of telomere elongation helicase 1; *RTEL1*-*TNFRSF6B*, RTEL1-TNFRSF6B readthrough (NMD candidate); *DLGAP1*, DLG-associated protein 1; *CGRRF1*, cell growth regulator with ring finger domain 1

**Table 2:** Allele frequencies for the AMD-associated variants identified with ROADTRIPS testing of Amish families in outbred populations (IAMDGC and gnomAD release 2.1)

Variant		Allele frequency		
		IAMDGC		gnomAD
rsID	Chromosome	Advanced AMD cases	Controls	Non-Finnish Europeans
rs200437673	9	0.004118	0.0043691	0.004341
rs151214675	20	0.00024779	0.00016824	0.0001936
rs140250387	18	0.001146	0.0012338	0.001611
rs115333865	14	0.019112	0.019993	0.01879

AMD, age-related macular degeneration; ROADTRIPS, RObust Association-Detection Test for Related Individuals with Population Substructure; IAMDGC, International Age-related Macular Degeneration Genomics Consortium; gnomAD, Genome Aggregation Database

### Linkage analysis

**Fig. 3:**
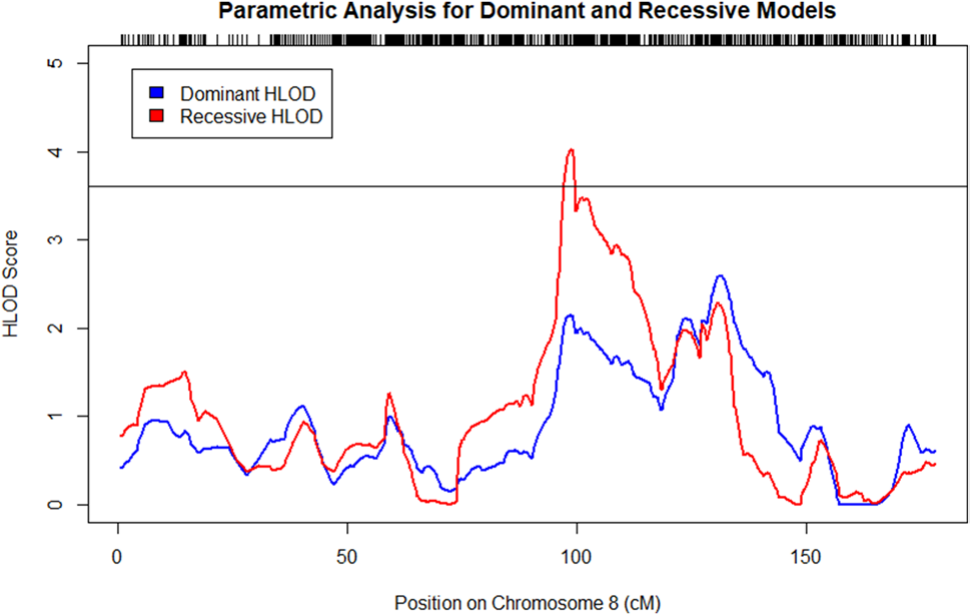
HLOD scores obtained from multipoint linkage analysis in MERLIN under dominant and recessive models on chromosome 8. The black line denotes genome-wide significance (HLOD Score > 3.6). A genome-wide significant HLOD score of 4.03 was observed under the recessive model. Tick marks along the upper *x*-axis correspond to the marker positions

**Fig. 4:**
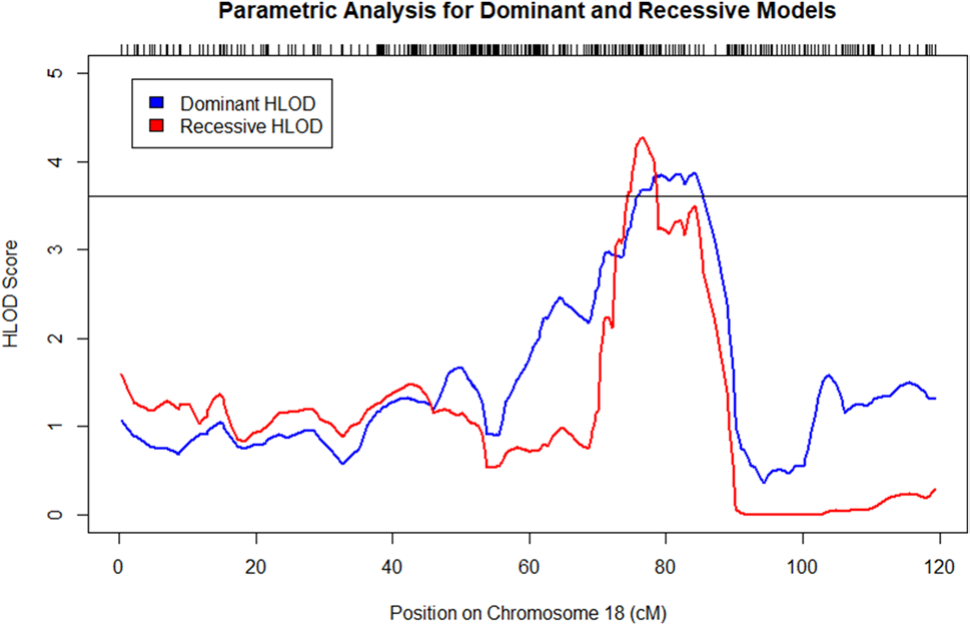
HLOD scores obtained from multipoint linkage analysis in MERLIN under dominant and recessive models on chromosome 18. The black line denotes genome-wide significance (HLOD Score > 3.6). The maximum dominant HLOD score was 3.87. The maximum recessive HLOD score was 4.27. Tick marks along the upper *x*-axis correspond to the marker positions

Multipoint linkage analyses under autosomal dominant and recessive models were performed to identify genomic loci linked to AMD in 16 Amish sub-pedigrees (Supplemental Figs. 7, 8; Supplemental Tables 5, 6). Under the recessive model with a disease allele frequency of 0.10, we identified genome-wide significant signals (HLOD > 3.6) on chromosome 8 (Table 3; Fig. 3). We also identified significant signals on chromosome 18 under the dominant and recessive models (Table 3; Fig. 4). These signals were robust to varying sub-pedigree structure (Supplemental Figs. 9, 10). Although we initially observed genome-wide significant signals (HLOD > 3.6) on chromosome 2 under the dominant and recessive models (Supplemental Table 7; Supplemental Fig. 11) and on chromosome 15 under the recessive model (Supplemental Table 7; Supplemental Fig. 12), these signals greatly reduced when we re-performed our analyses with different sub-pedigree structures (Supplemental Figs. 13, 14).

**Table 3:** Significant linkage loci identified from model-based multipoint linkage analyses of Amish families with disease allele frequency of 0.10

Model	Chromosome	Peak HLOD score	Region with HLOD > 3.6 (cM)	1-HLOD support interval (cM)
Recessive	8	4.03	97.28–99.61	96.745–105.65
Dominant	18	3.87	75.82–85.41	70.88–87.23
Recessive	18	4.27	74.39–78.51	74.025–78.87
				81.31–82.18
				83.46–84.80

The peak HLOD score is the maximum HLOD score obtained for the designated chromosome

HLOD, heterogeneity LOD; cM, centimorgans

**Fig. 5:**
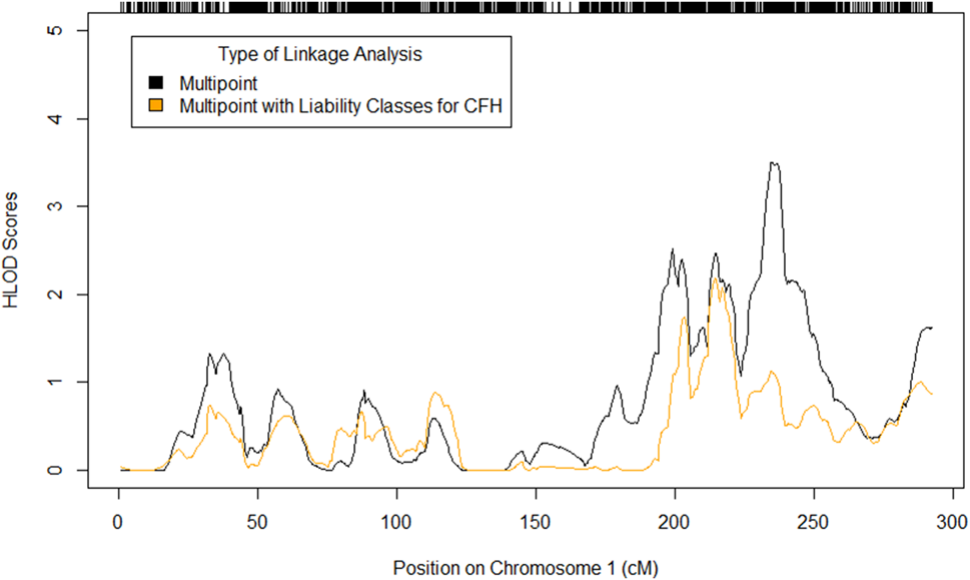
Conditional linkage analysis on chromosome 1 taking into account the Y402H and P503A carrier statuses. The black line denotes the HLOD scores from the multipoint linkage analysis performed without liability classes for the *CFH* variants. The orange line represents the distribution of HLOD scores from the conditional linkage analysis. The peak HLOD score in the unconditioned analysis was 3.503 at 234.6 cM (212,619,339 bp, build 37). The peak HLOD score in the conditioned analysis was 2.1836 at 214.47 cM (197,070,697 bp, build 37). The *CFH* gene boundaries are 196,621,008–196,716,634 bp (Ensembl, build 37)

In our multipoint linkage analysis under the dominant model with a disease allele frequency of 0.10, we observed a region on chromosome 1 that was nearly genome-wide significant (maximum HLOD = 3.50 at 234.60 cM; Supplemental Table 5). The peak of this region is about 16 MB from the complement factor H (*CFH*) gene, which has been repeatedly associated with AMD risk in the general population (Fritsche et al. 2016). We performed a conditional linkage analysis on the markers on chromosome 1 with liability classes for carriers of two AMD risk variants in *CFH* (Y402H and P503A) to determine whether these markers were driving the peak linkage signal on chromosome 1. Of the 86 affected individuals in the linkage analysis, 8 carry at least one copy of the P503A variant, and 63 have at least 1 copy of the surrogate for Y402H. Three of the P503A carriers are also heterozygous for the surrogate of Y402H. Our conditional linkage analysis on chromosome 1 using distinct classes for carriers of the surrogate for *CFH* Y402H and/or the *CFH* P503A variant demonstrated that the *CFH* locus was contributing to the peak signal we observed in the unconditioned analysis (Fig. 5).

### In silico functional analysis of genes from 1-HLOD support intervals of linkage regions

**Fig. 6:**
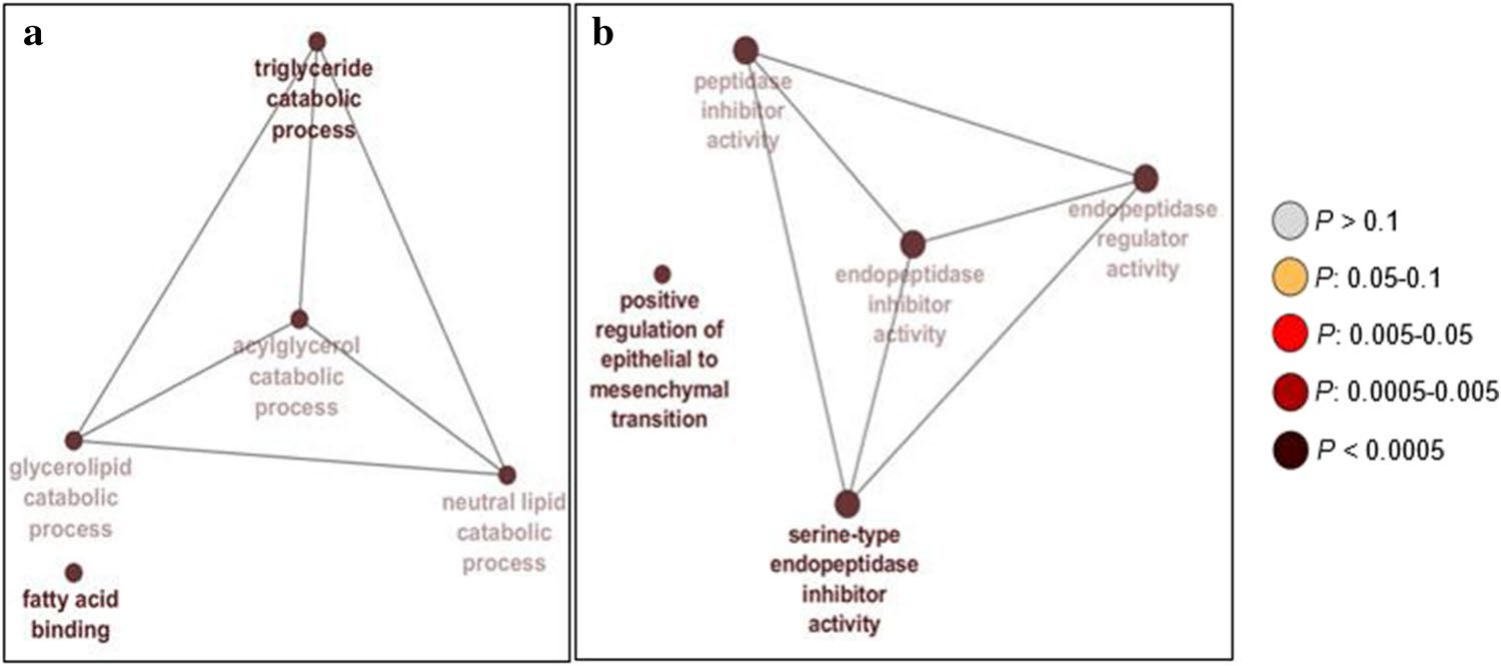
Gene Ontology (GO) Enrichment Networks for Genes from 1-HLOD Support Intervals from **a** chromosome 8 and **b** chromosome 18. The size of the nodes in each network illustrates the number of mapped genes to the depicted GO term, and the color of the node shows the significance of the term in each enrichment analysis. The leading term of each GO group is depicted in bold and represents the most significant ontology from the group. Full descriptions of these terms are available in Supplemental Tables 8 and 9

We performed GO enrichment analyses using right-sided hypergeometric tests on the genes from the 1-HLOD support intervals our linkage regions to identify any functional relationships through GO terms. *p* values were corrected for multiple testing using the Benjamini–Hochberg method. The region we identified with significant evidence of linkage on chromosome 8q21.11–q21.13 includes 19 genes (Supplemental Table 2). The 1-HLOD support interval of this peak contains 80 unique genes (Supplemental Table 2), including those that are involved in fatty acid binding and triglyceride catabolic processes (Fig. 6a; Supplemental Table 8). The region we found with significant evidence of linkage on chromosome 18q21.2–21.32 under the dominant model includes 47 genes (Supplemental Table 3). The 1-HLOD support interval of this peak contains 102 unique genes (Supplemental Table 3), including those that are implicated in serine-type endopeptidase inhibitor activity and positive regulation of epithelial to mesenchymal transition (Fig. 6b; Supplemental Table 9). The significant linkage region we observed on chromosome 18q21.2–21.31 under the recessive model includes 19 genes (Supplemental Table 4). The 1-HLOD support interval of this peak contains 46 unique genes (Supplemental Table 4), which were not overrepresented in any particular GO terms in our enrichment analysis. When we analyzed all the genes from our 1-HLOD support intervals on chromosome 8 and 18 together in ClueGO, the same GO terms from our chromosome-specific analyses were identified.

## Discussion

We performed association and linkage analyses on Amish families from Ohio and Indiana to uncover novel variants and loci for AMD. Using ROADTRIPS and kinship coefficients derived from the relationships detailed in the all-connecting path pedigree, we identified four novel variants associated with AMD in the Amish. Since we analyzed exome chip data, we recognize that these variants may not be the functional variants underlying these loci and that we might be observing their association signals because they are in linkage disequilibrium with the true functional variants for AMD. These four variants are independent and physically distant from the 52 AMD-associated variants identified by the IAMDGC (Fritsche et al. 2016). Association signals were not detected in the 5 directly genotyped variants in the *LCN9* gene on chromosome 9 or the 27 directly genotyped variants in the *RTEL1/RTEL1*-*TNFRSF6B* locus on chromosome 20 from the IAMDGC GWAS for advanced AMD. However, a few nominal signals (*p *< 0.05) were found in the IAMDGC loci for *DLGAP1* (*n* = 3 out of 144) on chromosome 18 and *CGRRF1* (*n* = 1 out of 6) on chromosome 14 (Fritsche et al. 2016). These three *DLGAP1* variants are not in linkage disequilibrium (LD) with one another (*r*^2^ < 0.2) but are within 500 KB of the variant we identified in the Amish. The *CGRRF1* variant that achieved a *p* value less than 0.05 in the IAMDGC GWAS and the variant we identified with ROADTRIPS in the Amish are 500 bp apart but are not in LD (*r*^2^ = 0.0001 in CEU population). The variant we found on chromosome 9 (rs200437673) is about 36.6 MB, 65.1 MB, 61.9 MB, and 30.9 MB away from the lead variants identified by the IAMDGC on this chromosome (rs1626340, rs71507014, rs10781182, and rs2740488, respectively) (Fritsche et al. 2016). The variant we identified on chromosome 20 (rs151214675) is about 5.6 and 17.7 MB away from the lead variants from the IAMDGC GWAS on chromosome 20 (rs201459901 and rs142450006, respectively) (Fritsche et al. 2016). The IAMDGC did not identify any AMD-associated variants on chromosome 18 (Fritsche et al. 2016). The lead variant in the AMD locus identified by the IAMDGC on chromosome 14 (rs61985136) (Fritsche et al. 2016) is almost 14 MB away from the variant we identified in the Amish (rs115333865). The four variants we identified in the Amish are rare to low-frequency in the IAMDGC data and the gnomAD database. While allele frequencies for these variants are fairly similar in advanced AMD cases and controls from the IAMDGC, these variants were significantly associated with AMD in our Amish cohort. It was previously demonstrated that the Amish population has a lower genetic burden of known AMD variants (Hoffman et al. 2014); therefore, this may suggest that the Amish have different etiology for AMD than the general population of European descent.

Of the AMD-associated variants that we identified in this study, only the *RTEL1* variant (rs151214675) is cataloged in the ClinVar database, but it is considered a variant of unknown significance (https://www.ncbi.nlm.nih.gov/clinvar/RCV000504185/). This variant also maps to the naturally occurring *RTEL1*-*TNFRSF6B* read-through transcript of this locus, which is a candidate for nonsense-mediated mRNA decay and unlikely to yield a protein product. *RTEL1* encodes a regulator of a telomere elongation helicase, which maintains telomere length and genomic stability (Barber et al. 2008; Codd et al. 2013; Ding et al. 2004). Telomere length has been hypothesized as a marker of aging because telomeres shorten with age and the presence of short telomeres directs the cell to enter senescence (von Zglinicki and Martin-Ruiz 2005). Oxidative stress can also contribute to the reduction of telomere length in cells and has been characterized as a contributing factor to AMD pathophysiology (Shaw et al. 2016; von Zglinicki and Martin-Ruiz 2005). Lipocalins constitute a family of extracellular proteins that are responsible for transporting small lipids such as fatty acids, retinoids, and steroids (Suzuki et al. 2004). There are no *LCN9* variants documented in the GWAS Catalog (https://www.ebi.ac.uk/gwas/). However, another lipocalin family member (lipocalin-2, *LCN2*) may modulate inflammation in retinal degeneration by promoting cell survival responses and regulating the production of inflammatory proteins (Parmar et al. 2018). Additionally, tear lipocalins constitute a large group of lipid-binding proteins in tears and may serve as potential biomarkers for diabetic retinopathy and Alzheimer’s disease (Kallo et al. 2016; Wang et al. 2017). *DLGAP1* encodes a guanylate kinase-associated protein involved in protein–protein interactions with scaffold proteins in the post-synaptic density of excitatory synapses (Feng and Zhang 2009; Li et al. 2016). *DLGAP1* also is one of nine genes located in a chromosomal region (myopia-2, MYP2) that demonstrated significant genetic linkage with autosomal dominant high myopia (Scavello et al. 2005; Young et al. 1998). The proteins encoded by *DLGAP1* and lipocalin genes (*LCN1* and *LCN2)* were also found to be expressed in the human choroid-retinal pigment epithelial complex (Skeie and Mahajan 2014). The variant we identified in *DLGAP1* is about 3 MB away from an AMD variant (rs9973159) identified in a genome-wide association study accounting for age-stratified effects in the IAMDGC data (Winkler et al. 2018). *CGRRF1* encodes a cell growth regulator that has been associated with eye morphology in humans (Lee et al. 2017). While the protein product of this gene is not well-characterized, it has proposed as a modulator of Evi, which is a transmembrane protein involved in Wnt protein secretion (Glaeser et al. 2018). Canonical Wnt signaling has been implicated in retinal inflammation and may have a role in AMD pathology (Zhou et al. 2010). Additional studies will be required to elucidate the roles these genes might have in AMD etiology.

In our linkage screens in the Amish, we identified a novel susceptibility locus for AMD on chromosome 8q21.11–q21.13 under our recessive model. This region does not overlap with AMD loci previously identified with genome-wide linkage screens, which occur on chromosomes 8p21 and 8q11.2 (Seddon et al. 2003). In their recent GWAS, the IAMDGC identified a susceptibility locus for AMD on chromosome 8p21.3 with the most significant signals coming from rs13278062 and rs79037040 in the *TNFRSF10A/LOC389641* and *TNFRSF10A* genes (Fritsche 2013, 2016). In our linkage analyses of chromosome 8, we observed a maximum HLOD score of 0.67 in this gene region under the recessive model with disease allele frequency of 0.10. The strongest single variant *p* value observed by the IAMDGC in our linkage region was 6.54 × 10^−4^, which does not reach classical GWAS significance (*p* < 5 × 10^−8^). Common variants associated with optic nerve degeneration in glaucoma have also been identified in 8q22, which is near our linkage region (Wiggs et al. 2012).

Based on our GO enrichment analysis with ClueGO, the genes from the 1-HLOD support interval of our significant linkage region on chromosome 8q21.11–q21.13 have functional annotations related to triglyceride catabolic processes and fatty acid binding. Components of lipid metabolism have been previously implicated in the genetic etiology and pathophysiology of AMD. Lipids comprise about 40% of the composition of drusen, which are a hallmark of AMD (Wang et al. 2010). In their most recent GWAS, the IAMDGC determined that several lipid pathways from the Reactome and GO pathway databases were enriched for genes from the 34 AMD susceptibility loci they identified (Fritsche et al. 2016). Known AMD-associated genes (*APOE* and *LIPC*) (Fritsche et al. 2016) are also described as members of the triglyceride, neutral lipid, acylglycerol, and glycerolipid catabolic processes (GO:0019433, GO:0046461, GO:0046464, and GO:0046503, respectively), which were enriched in genes from our linkage region. Additionally, although consuming high amounts of saturated and monounsaturated fats has been associated with AMD risk (Delcourt et al. 2007), dietary intake of omega-3-fatty acids has been attributed to reducing the risk of AMD (Delcourt et al. 2007; SanGiovanni and Chew 2005; SanGiovanni et al. 2008; Seddon et al. 2006a). There is not a consistent association between triglyceride levels and AMD risk. Some studies observed lower triglyceride levels in patients with early AMD (Klein et al. 2003), the choroidal neovascularization subtype of advanced AMD (Merle et al. 2014), or any type of AMD (Paun et al. 2015; Roh et al. 2008; Semba et al. 2014). However, other studies found higher triglyceride levels were associated with AMD status (Munch et al. 2013; Nowak et al. 2005). Recently, levels of triglycerides were associated with a decreased risk for AMD and smaller drusen area (Colijn et al. 2018). Further functional studies would need to be performed to definitively implicate the role of fatty acid binding and triglyceride levels in AMD pathology.

From our multipoint linkage analyses with dominant and recessive models of inheritance, we identified novel AMD loci on chromosome 18q21.2–21.32 and 18q21.2–21.31, respectively. These regions do not overlap with the AMD-associated variant (rs140250387) we identified in our association test. Although both analyses interrogate the genetic etiology of the trait, linkage analyses identify chromosomal segments co-segregating with traits in families (Morton 1955); whereas, association analyses compare allele frequencies between individuals with and without the trait of interest (Hirschhorn and Daly 2005). Therefore, their results can be independent of one another. The IAMDGC did not identify any AMD susceptibility loci on chromosome 18 in either of their recent GWAS, and another study using IAMDGC data identified an AMD gene × age interaction on chromosome 18p, which is independent of our linkage peak (Fritsche 2013, 2016; Winkler et al. 2018). The most notable single-marker *p* value observed in the IAMDGC GWAS was 1.27 × 10^−4^, which occurred for a variant that falls within our linkage regions under both dominant and recessive models. Previous GWAS have found suggestive AMD variants on chromosomes 18q22.1 (Naj et al. 2013) and 18q23 (Sobrin et al. 2012), which are located outside of the 1-HLOD support interval of our linkage region. The C allele of the 18q22.1 variant (rs17073641) has a strong protective effect in never smokers but increases the risk of AMD in smokers (Naj et al. 2013). The 18q23 variant (rs1789110), which occurs near the myelin basic protein (*MBP*) gene, demonstrated suggestive evidence of association with geographic atrophy (Sobrin et al. 2012).

The 1-HLOD support interval of our significant linkage region on chromosome 18q21.2–21.32 contains genes that have functional annotations in GO processes such as positive regulation of epithelial to mesenchymal transition (EMT) and serine-type endopeptidase inhibitor activity. Another known AMD risk gene, *TGFBR1*, (Fritsche 2013, 2016) has been previously described as a part of the positive regulation of EMT (GO:0010718). The EMT of retinal pigment epithelial (RPE) cells is considered one of several biological processes that are responsible for the formation of subretinal fibrosis in the macula following choroidal neovascularization in advanced AMD (Ishikawa et al. 2016a). EMT has also been proposed as a mechanism employed by RPE cells to survive in the stressful macular microenvironment during dry AMD progression (Ghosh et al. 2018). Therefore, it has been suggested that therapeutically targeting RPE cells in EMT may help treat patients with advanced AMD and subretinal fibrosis (Ghosh et al. 2018; Ishikawa et al. 2016b; Kobayashi et al. 2019). Several of the serine proteinase inhibitor (serpin) family members from the 1-HLOD support interval have also been described in the context of AMD-related pathologies. The proteins encoded by *SERPINB5* (maspin) and *SERPINB13* (headpin) are known for their anti-angiogenic properties, which may have implications for the choroidal neovascularization subtype of advanced AMD (Pescosolido et al. 2014; Shellenberger et al. 2005; Zhang et al. 2000). A genetic variant near *SERPINB2* was identified as a risk factor for AMD in smokers and a protective factor in nonsmokers in gene-environment interaction analyses (Naj et al. 2013). Another gene from our 1-HLOD support interval, *TCF4*, has repeatedly been association with Fuchs endothelial dystrophy, which is characterized by the deterioration of the corneal epithelium (Baratz et al. 2010; Kuot et al. 2012; Li et al. 2011; Riazuddin et al. 2011; Thalamuthu et al. 2011). *C3* and *TIMP3* are known AMD-associated genes identified by the IAMDGC that are also described in the following GO terms from our analyses: endopeptidase regulator activity (GO:0061135), peptidase inhibitor activity (GO:0030414), and endopeptidase inhibitor activity (GO:0004866). Additionally, the only subtype-specific variant identified for the CNV subtype of advanced AMD was located near the *MMP9* gene on chromosome 20, which is a member of the endopeptidase family that participates in extracellular matrix degradation (Fritsche et al. 2016).

Although the major peak in our initial linkage analysis on chromosome 1 was diminished in our conditional linkage analysis, a suggestive linkage peak (HLOD ~ 2) remained around 213–217 cM. This genomic region corresponds to a locus of approximately 5 MB that contains multiple genes including *CFH* and *CFHR1*-*5.* These genes are located within the regulation of complement activation (RCA) gene cluster on chromosome 1 and encode members of the factor H/CFHR family of proteins (Male et al. 2000). Polymorphisms and deletions in *CFH* and *CFHRs* have been previously associated with AMD (Edwards et al. 2005; Fritsche et al. 2016; Hageman et al. 2005; Haines et al. 2005; Klein et al. 2005; Lores-Motta et al. 2018; Martinez-Barricarte et al. 2012), including rare variants in *CFH* (Hoffman et al. 2014; Raychaudhuri et al. 2011) and a protective deletion of *CFHR1* and *CFHR3* (Fritsche et al. 2010; Hageman et al. 2006; Hughes et al. 2006). More detailed mapping of this region in our cohort will be necessary to determine which polymorphisms in these gene(s) are contributing to our linkage signal.

In this study, we performed association and linkage screens in Amish families from Ohio and Indiana. We identified four novel rare variants associated with increased AMD risk in the Amish and novel susceptibility loci on chromosomes 8q21.11–q21.13 (maximum recessive HLOD = 4.03) and 18q21 (maximum dominant HLOD = 3.87; maximum recessive HLOD = 4.27). These findings suggest novel genetic factors for AMD in the Amish and demonstrate the benefits of analyzing the genetic architecture of a complex trait, like AMD, in closely related individuals from an isolated population. While family-based studies do not require sample sizes as large as traditional GWAS, the sample size for this study (*n* = 175) limited its power to detect novel AMD variants and loci. This could be remedied with the ascertainment of additional members of many Amish communities. Additionally, more genetic data could be obtained from whole exome sequencing or dense genotyping for a panel such as the Multi-Ethnic Genotyping Array (MEGA) of about 2 million exonic markers for GWAS and ancestry-specific variants. Additional studies are needed to replicate their associations and validate their roles in the development or progression of AMD in outbred populations. Analyses could also be performed for the subtypes or endophenotypes of AMD to identify subtype-specific trends or associations with other binary and quantitative traits that may be pertinent for AMD etiology. Such studies may help resolve the missing heritability of AMD and provide novel insights to AMD pathophysiology.

## Electronic supplementary material

Below is the link to the electronic supplementary material.

Supplementary material 1 (DOCX 845 kb)

Supplementary material 2 (XLSX 9 kb)

Supplementary material 3 (XLSX 11 kb)

Supplementary material 4 (XLSX 12 kb)

Supplementary material 5 (XLSX 10 kb)

Supplementary material 6 (XLSX 10 kb)

Supplementary material 7 (XLSX 9 kb)

Supplementary material 8 (XLSX 8 kb)

Supplementary material 9 (XLSX 10 kb)

Supplementary material 10 (XLSX 10 kb)
