# Supplementary material for: Rare variants and loci for age-related macular degeneration in the Ohio and Indiana Amish
Source: Hum Genet. 2019 Jul 31;138(10):1171–82. doi: 10.1007/s00439-019-02050-4 (PMC6745026; doi:10.1007/s00439-019-02050-4)
Supplement: Supplementary file 1 — Supplementary material 1 (DOCX 845 kb) [file 439_2019_2050_MOESM1_ESM.docx]

**Rare variants and loci for age-related macular degeneration in the Ohio and Indiana Amish**

Andrea R. Waksmunski^1,2,3^, Robert P. Igo, Jr.^3^, Yeunjoo E. Song^3^, Jessica N. Cooke Bailey^2,3^, Renee Laux^3^, Denise Fuzzell^3^, Sarada Fuzzell^3^, Larry D. Adams^4^, Laura Caywood^4^, Michael Prough^4^, Dwight Stambolian^5^, William K. Scott^4^, Margaret A. Pericak-Vance^4^, and Jonathan L. Haines^1,2,3*^

^1^Department of Genetics and Genome Sciences, Case Western Reserve University, Cleveland, Ohio, U.S.A.

^2^Cleveland Institute for Computational Biology, Case Western Reserve University, Cleveland, Ohio, U.S.A.

^3^Department of Population and Quantitative Health Sciences, Case Western Reserve University, Cleveland, Ohio, U.S.A.

^4^John P. Hussman Institute for Human Genomics, University of Miami Miller School of Medicine, Miami, Florida, U.S.A

^5^Department of Ophthalmology, University of Pennsylvania, Philadelphia, Pennsylvania, U.S.A.

^*^Correspondence should be addressed to J.L.H. ([jonathan.haines@case.edu](mailto:jonathan.haines@case.edu)).

**Supplemental Fig. 1**

Outline of quality control steps. Genotype calling and clustering, quality control, and manual rare variant calling were performed in GenomeStudio. Rare variant calling was also performed separately in zCall. Variants with 4 more heterozygous calls in zCall were flagged for manual review in GenomeStudio. Additional quality control measures were utilized with PLINK, and monomorphic variants were excluded from downstream analyses. The color scheme indicates the software tool used at that step


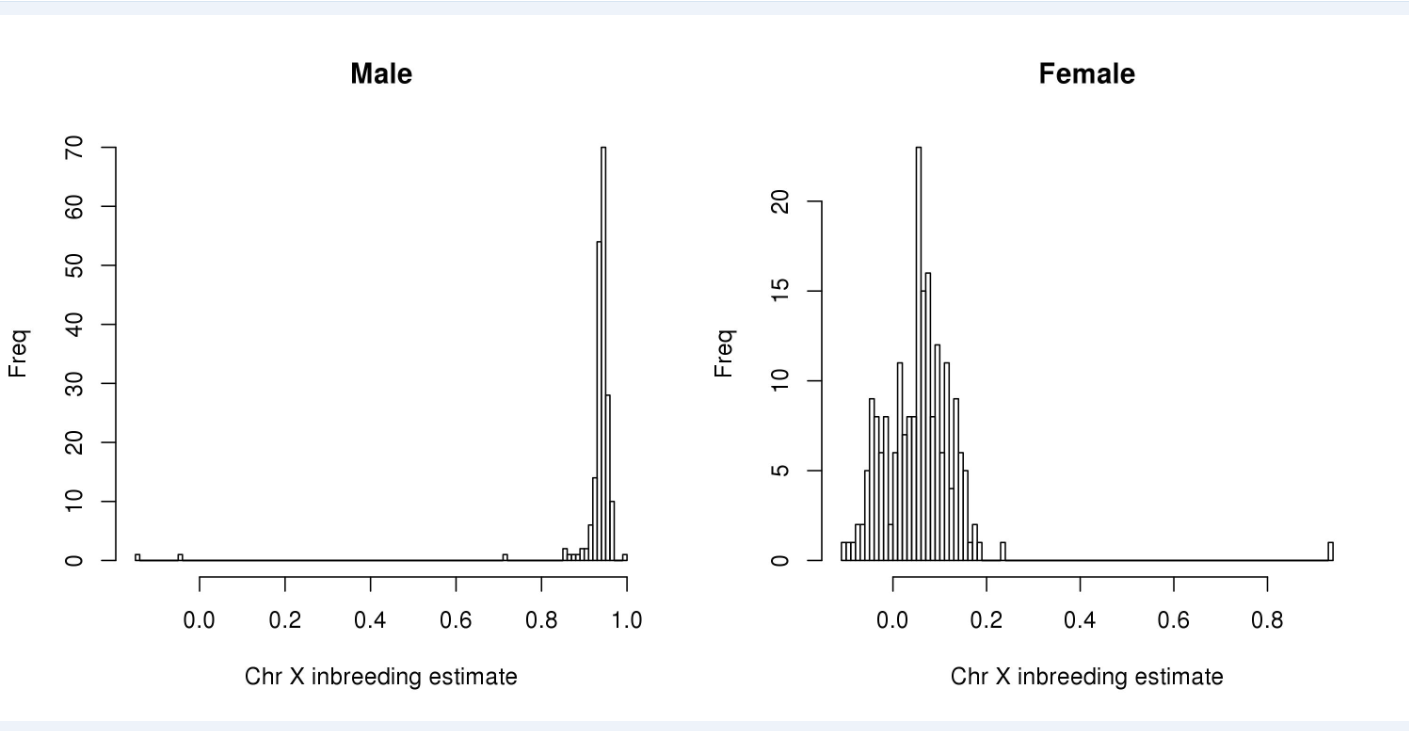


**Supplemental Fig. 2**

Sex mismatch determined for 2,466 post-quality control samples. Male samples are expected to have values above 0.8, and females are expected to have values less than 0.2. One true sex mismatch in our Amish samples was identified and removed from subsequent analyses. Other apparent sex mismatches were among the non-Amish samples, which were all excluded from our association and linkage analyses


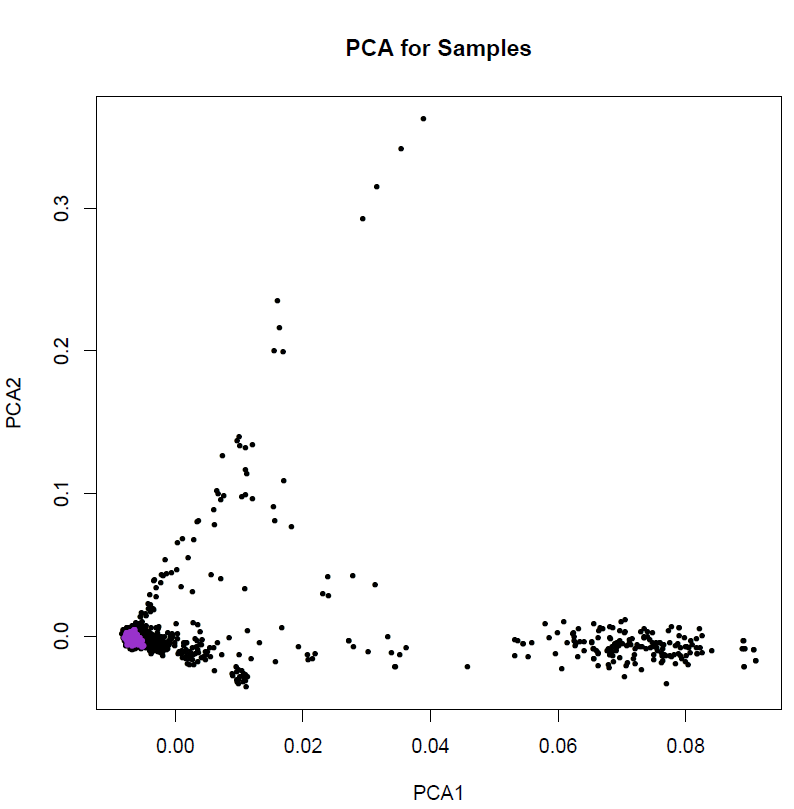


**Supplemental Fig. 3**

Principal components analysis demonstrating race distribution for the 2,466 post-QC samples. Our Amish samples are highlighted in purple


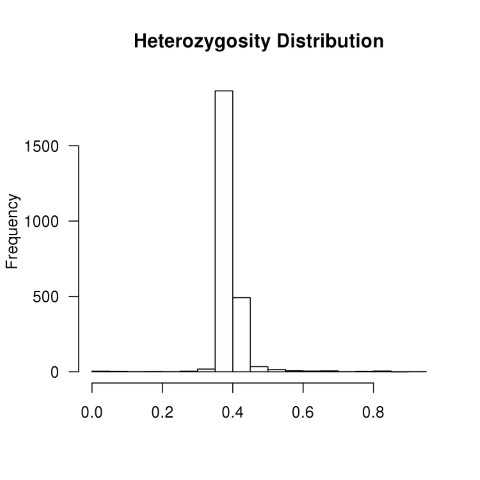


Heterozygosity

**Supplemental Fig. 4**

Heterozygosity distribution of the 2,466 samples. Few samples had heterozygosity values greater than 0.8 indicating them as outliers. None of the Amish samples used in this study had heterozygosity values in this range

**Supplemental Fig. 5**

All-connecting path pedigree of the 180 Amish individuals genotyped on the exome chip drawn using the Pedigraph software tool and information from the Anabaptist Genealogy Database (AGDB). Circles represent females, and squares represent males. The colored lines connect children to their parents. The five individuals we omitted from our analyses due to extensive distant relatedness are surrounded by the blue box


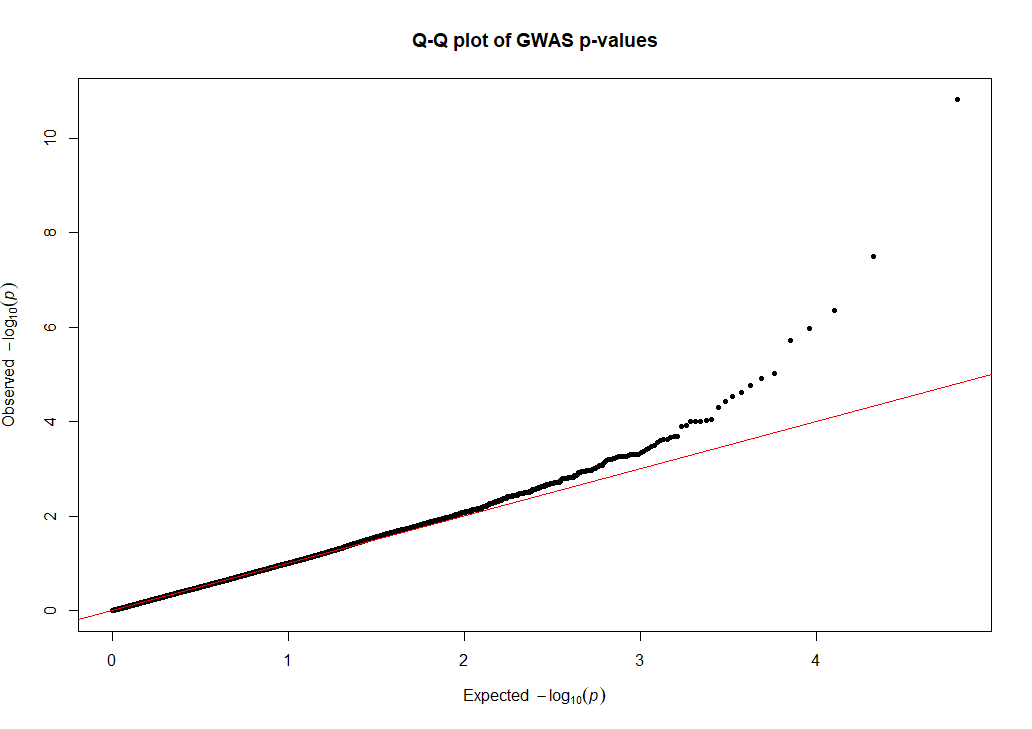


**Supplemental Fig. 6**

Quantile-Quantile (QQ) plot of *p-*values obtained from association testing using ROADTRIPS. *P*-values were obtained from the RM test in ROADTRIPS. The genomic control parameter was 1.05


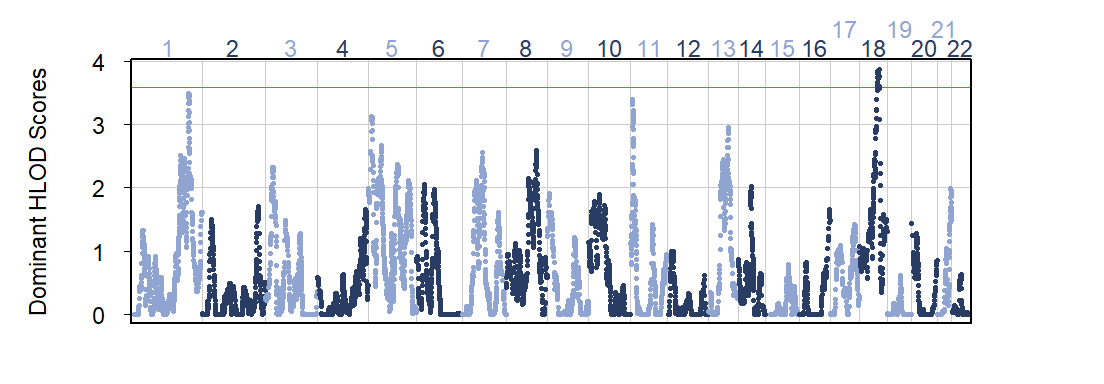


**a**


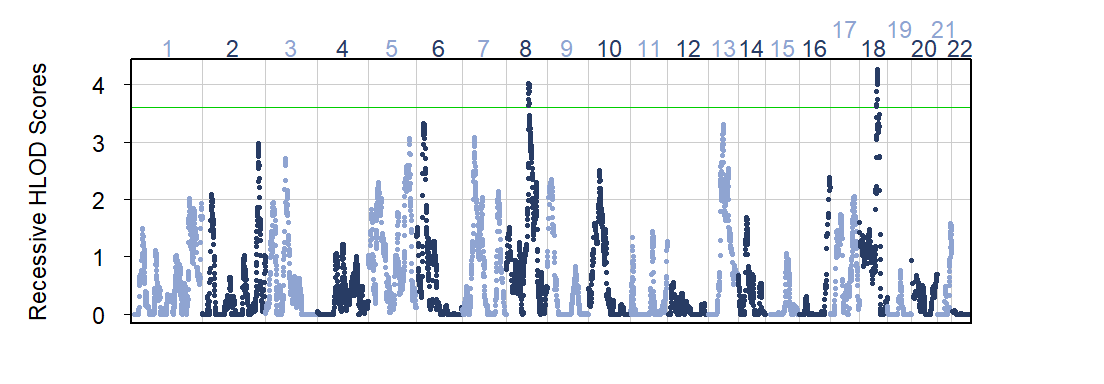


**b**

**Supplemental Fig. 7**

Genome-wide HLOD scores for the autosomes under affecteds-only (a) dominant and (b) recessive models with disease allele frequency of 0.10. The green line in each plot designates the genome-wide significance threshold (HLOD > 3.6)


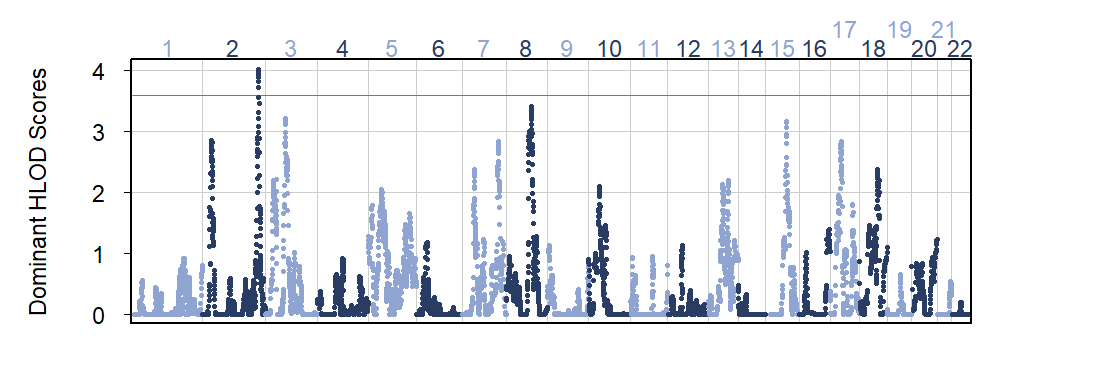


**a**


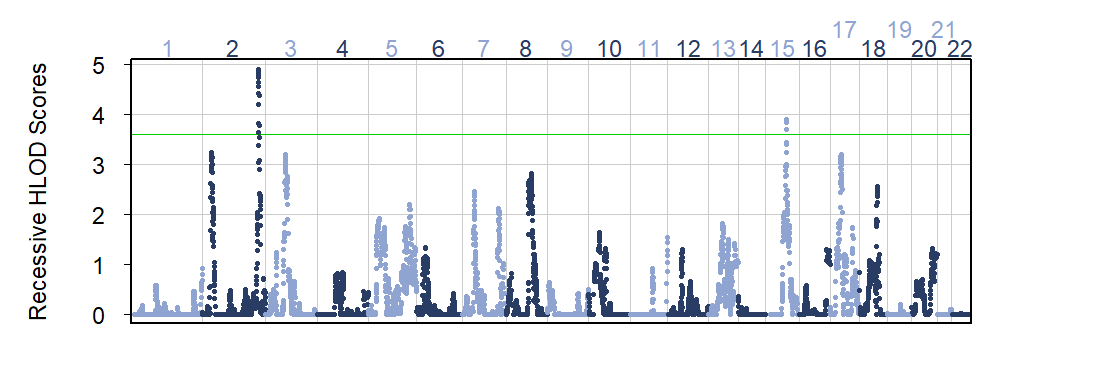


**b**

**Supplemental Fig. 8**

Genome-wide HLOD scores for the autosomes under affecteds-only (a) dominant and (b) recessive models with disease allele frequency of 0.01. The green line in each plot designates the genome-wide significance threshold (HLOD > 3.6)


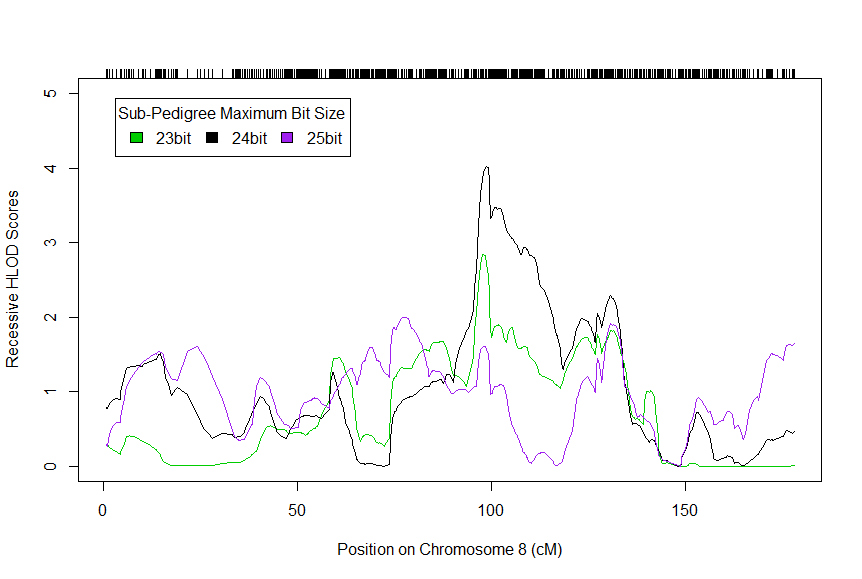


**Supplemental Fig. 9**

HLOD scores for chromosome 8 under the recessive model assuming risk allele frequency of 0.10 with different sub-pedigree structures. Linkage peaks were as follows for the maximum pedigree sizes tested: 23 bits, 98.01 cM (HLOD = 2.84); 24 bits, 98.80 cM (HLOD = 4.027); 25 bits, 77.53 cM (HLOD = 2.00)


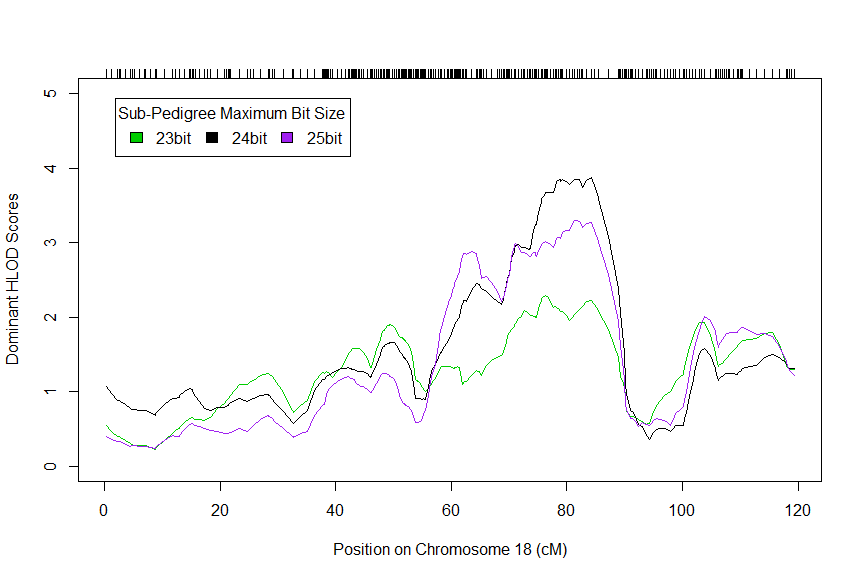


**a**


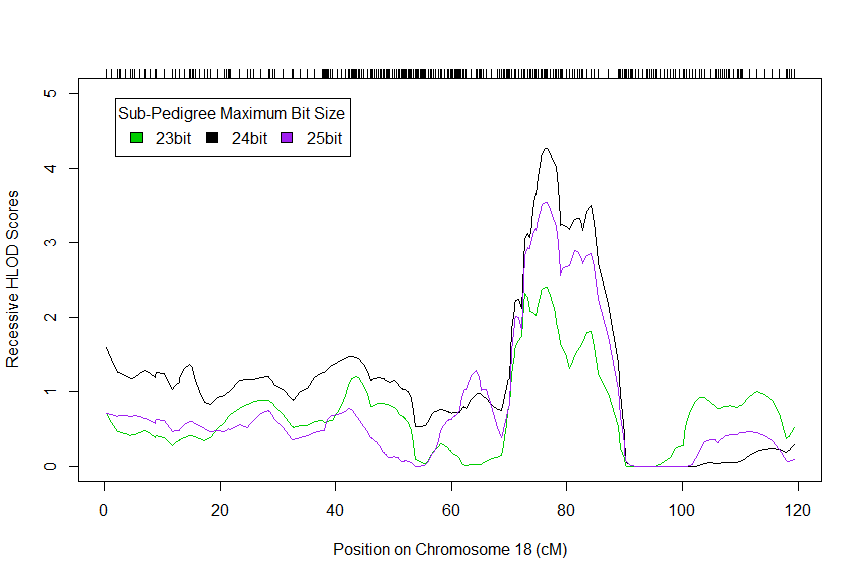


**b**

**Supplemental Fig. 10**

HLOD scores for chromosome 18 under (a) dominant and (b) recessive models assuming disease allele frequency of 0.10 with different sub-pedigree structures. Linkage peaks from the dominant model were as follows for the maximum pedigree sizes tested: 23 bits, 76.38 cM (HLOD = 2.29); 24 bits, 84.18 cM (HLOD = 3.87); 25 bits, 81.31 cM (HLOD = 3.30). Linkage peaks from the recessive model were as follows for the maximum pedigree sizes tested: 23 bits, 76.38 cM (HLOD = 2.40); 24 bits, 76.38-76.50 cM (HLOD = 4.27); 25 bits, 76.38 cM (HLOD = 3.54)


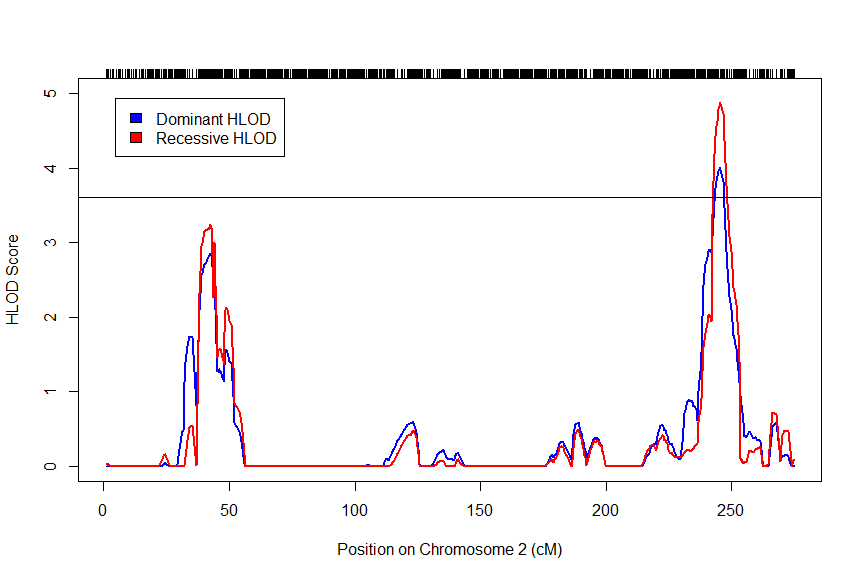


**Supplemental Fig. 11**

HLOD scores obtained from multipoint linkage analysis in MERLIN under the affecteds-only dominant and recessive models on chromosome 2. The black line denotes genome-wide significance (HLOD Score > 3.6). The maximum dominant HLOD score was 4.02, and the maximum recessive HLOD score was 4.89. Tick marks along the upper x-axis correspond to the marker positions


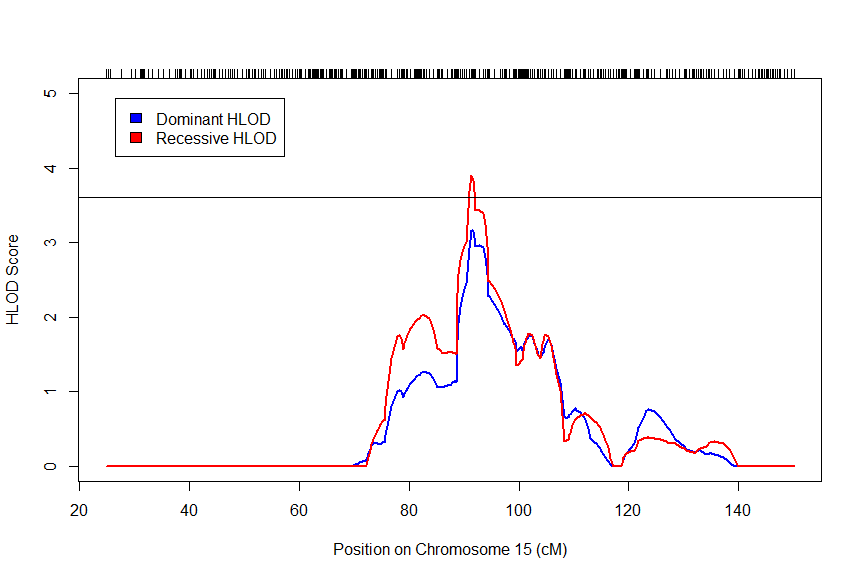


**Supplemental Fig. 12**

HLOD scores obtained from multipoint linkage analysis in MERLIN under the affecteds-only dominant and recessive models on chromosome 15. The black line denotes genome-wide significance (HLOD Score > 3.6). The maximum HLOD score was 3.90, which was obtained under the recessive model. Tick marks along the upper x-axis correspond to the marker positions


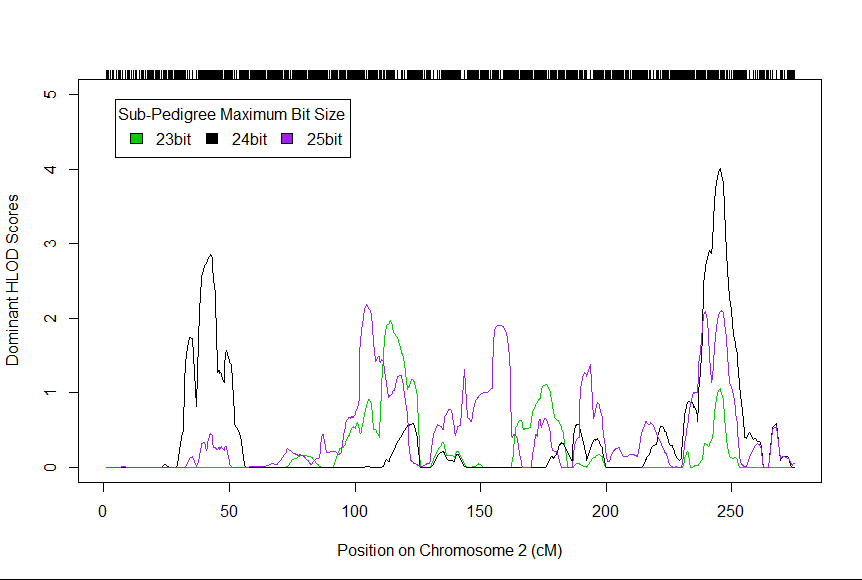

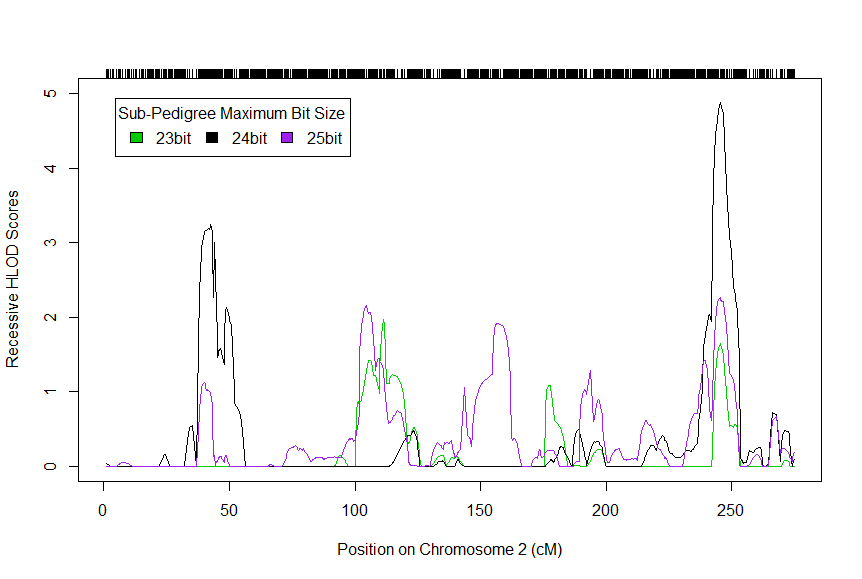


**a**

**b**

**Supplemental Fig. 13**

Disparate HLOD scores for chromosome 2 with different sub-pedigrees structures. HLOD scores were obtained for markers on chromosome 2 under **(a)** dominant and **(b)** recessive models assuming disease allele frequency of 0.01 with different sub-pedigree structures. Linkage peaks from the dominant model were as follows for the maximum pedigree sizes tested: 23 bits, 114.02 cM (HLOD = 1.97); 24 bits, 245.36 cM (HLOD = 4.02); 25 bits, 104.49 cM (HLOD = 2.18). Linkage peaks from the recessive model were as follows for the maximum pedigree sizes tested: 23 bits, 111.18 cM (HLOD = 1.97); 24 bits, 245.36 cM (HLOD = 4.89); 25 bits, 245.36 cM (HLOD = 2.27)


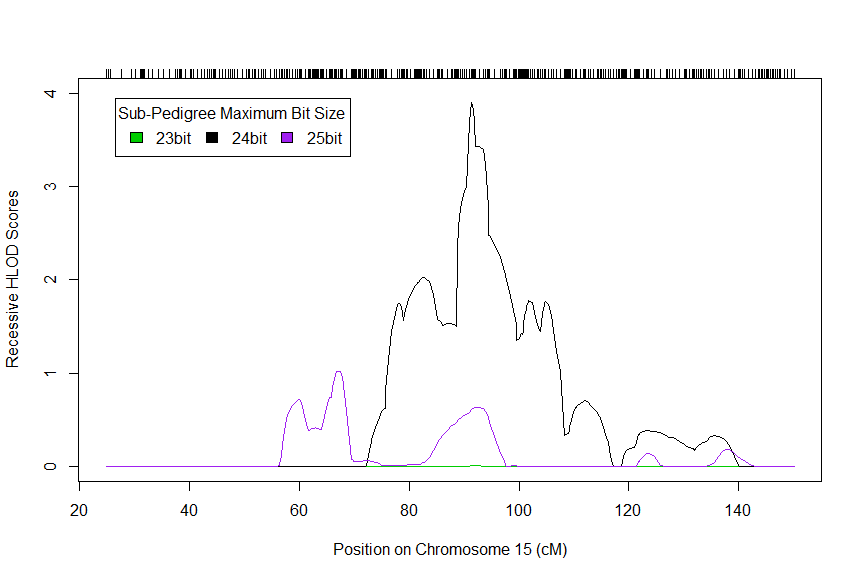


**Supplemental Fig. 14**

Disparate recessive HLOD scores for chromosome 15 with different sub-pedigrees structures. Parametric HLOD scores were calculated based on varying pedigree structures for markers on chromosome 15 under a recessive model. Linkage peaks from the recessive model were as follows for the maximum pedigree sizes tested: 23 bits, 91.69 cM (HLOD = 0.0056); 24 bits, 91.27 cM (HLOD = 3.90); 25 bits, 67.17 cM (HLOD = 1.02)
